# The microstructure, mechanical and electrochemical properties of 3D printed alloys with reusing powders

**DOI:** 10.1038/s41598-023-28971-9

**Published:** 2023-02-24

**Authors:** Mirjam Bajt Leban, Miha Hren, Tadeja Kosec

**Affiliations:** Slovenian National Building and Civil Engineering Institute, Dimičeva Ulica 12, Ljubljana, Slovenia

**Keywords:** Engineering, Materials science

## Abstract

CoCrMo and Ti6Al4V are widely used in medical, dental and 3D printing technology, allowing the accurate fabrication of geometrically complicated structures. In order to reduce the costs of printed objects, the reuse of powder is common daily practice. When using 3D printing technology, the direct impact of elevated temperatures and the influence of the laser beam may change the properties of the powder when it is reused, thus affecting the final properties of the printed object. The main aim of the present study was to investigate the impact of reused powder on the mechanical, microstructural and electrochemical properties of 3D printed objects. 3D printed objects fabricated from virgin and reused powder of both alloys were analyzed by metallographic observation, computed tomography, XRD and electrochemical methods. The main finding of the study was that the use of reused powder (recycled 3 times) does not detrimentally affect the mechanical and corrosion integrity of 3D printed CoCr and Ti6Al4V alloys, especially for the purpose of applications in dentistry.

## Introduction

The production of metal objects through additive manufacturing (AM) processes has rapidly expanded over the past few decades^1^. It proves to be most useful for the manufacture of small series objects of complicated geometries in the aerospace, energy and automotive industries, as well as in medicine^2–5^. AM is the opposite process to subtractive technologies, and involves joining powder materials layer upon layer to create objects from three-dimensional (3D) data models (ASTM F2792-12a: 2013 Standard Terminology For Additive Manufacturing Technologies). Theoretically, the amount of waste material when using AM can be zero, if the remaining residue powder, which was not used to build the object, is reused. With a single print, however, only 10 to 50% of the total amount of powder in the chamber is used, which largely depends on the size of the object printed^6^. Since the cost of powder could be over 10 times more expensive than the same weight of metal produced conventionally, there is great interest in reusing it. The final cost of the AM object therefore strongly depends on the possibility of reusing the powder^1,6,7^. This is even more pronounced in dentistry, where the elements produced by additive technologies are small, and the proportion between used and unused powder is high. There is considerable doubt concerning the use of recycled powder amongst users of dental products, primarily with regard to the impact on corrosion properties and biocompatibility, as well as on the mechanical properties of products.

During selective laser melting, the generation of laser spatters is commonly observed over a wide range of volumetric energy densities. This occurs due to recoil pressure, the Marangoni effect and the effect of heat in the molten pool. Laser spattering degrades the quality of printed parts for various reasons, including (i) a change in their chemical composition, (ii) the oxidation of powders, whereby more volatile elements are more sensitive at a given oxygen partial pressure, and (iii) balling. The most commonly observed defects in the printed structures are porosity and surface defects^8–10^. Powder particles positioned close to the printed object are exposed to heat and, consequently, they can be contaminated by oxygen and other gases^11,12^. These parameters affect the particle size distribution (PSD), chemical composition, surface appearance (roughness), microstructure and, through the powder flowability, the density of 3D printed objects^6,7,11–13^. A key step in reusing powder is its recycling procedure after each use, which, at the minimum, consists of sieving the powder through meshes of different sizes^14–19^. In addition, some more complex reconditioning methods can be applied^20^. Sieving removes any satellite particles that would impair the flowability of the powder. Results of some studies^14,16,18^ have shown that such recycling causes a narrowing of the particle size distribution (PSD) graph, while no narrowing was reported in another study^14^, only the PSD curves shifted upwards. In the studies previously mentioned^14,15,17,21^, however, an improvement was reported in the flowability of the reused powder. Larger agglomerates (satellite particles) removed during sieving might be the main reason for the narrowing of the PSD, and, together with the reduction of the humidity of the powder, also results in improved flowability and consequently the lower porosity^18^. It is assumed that reusing the powder would have an influence on its porosity^22^. The conclusions of previous studies are, however, contradictory in this regard. In the case of the nickel alloy INCONEL® alloy 718 (UNS N07718/W.Nr. 2.4668), only a slight increase in the porosity of a built object was found to be caused by using recycled powder^14^. In a different study^13^, the porosity was found to increase in the first few cycles of reusing the powder, but it later decreased. In other studies conducted on Ti6Al4V, powder recycling was reported to have no influence on the porosity^12^ and mechanical properties^23^ of the final object.

Microtomography (microCT), which is commonly used to study the porosity of AM parts in relation to their mechanical properties^24,25^, can also be used to study the influence of powder reuse on pore and particle size distribution. While some researchers have used microCT to study the porosity of reused powder^7,26^, Ghods et al.^27^ recently investigated the porosity of printed Ti6Al4V AM parts using microCT and found that over 30 printing cycles powder reuse had no significant impact on the total porosity, pore size distribution or sphericity of pores.

The efficiency of recycling also has a large impact on the properties of a 3D printed object. Some influences of exposing the powder to heat and gases in the printing atmosphere, such as oxygen uptake, and changes to the chemical composition and microstructure, cannot be restrained by recycling^15,22,28,29^. In order to achieve the target properties, it is therefore of great interest to understand the impacts of these factors on the properties of the final object. This is the main reason for many studies^6,13–18,20,21,30^ that investigate the properties of objects 3D printed with reused powder. The high cooling rate during the selective laser melting (SLM) AM process causes a change in the microstructure of the unused powder, which deviates from the microstructure of virgin powder (intermetallic Laves phases in Ni alloys, and α′ martensite in the Ti6Al4V alloy). No evidence was, however, found that such a microstructural change would have any influence on the microstructure of a 3D printed object^13,14^. The main change noticed when reusing the powder multiple times was an increase in the oxygen content of the powder, as well as that of the built object. This effect was most pronounced in Ti-alloys, where oxygen can easily diffuse into the lattice at elevated temperatures. Interstitial elements cause expansion of the lattice^31^, which results in increased hardness, yield and ultimate strength, as well as decreased ductility^32,33^.

The novelty of this paper is a special focus on three-time-used powder for the fabrication of dental objects and a holistic approach to the study of microstructural and corrosion properties and its effect on the porosity and hardness properties, which are important for applications in dentistry. Namely, the results from the published literature are related to multiple recycling cycles^13,15,16,27^, whereas the corrosion resistance of 3D printed objects made from new and recycled powders has not been yet compared. We focused on examining the properties of powder particles after a few cycles of reuse, as well as on the porosity and microstructural, mechanical and corrosion properties of a 3D printed structure intended for dental use. Powder that had been used three times was of special interest, since it is common practice to reuse powder this number of times in the technological process related to the production of dental parts, whereas results from the literature relate to powder that has been recycled more times^13,15–17,27,34^. In addition to the lack of results and information in the literature regarding the impact of using powder that has been recycled a few times, which is far more pronounced in CoCr than in a Ti6Al4V alloy, conflicting information on the effects on the properties of printed objects is also a major problem.

The first aim of our investigation was to find out if there are any differences between the properties of the virgin and reused powder examined, or between the objects fabricated from virgin and reused powder made from CoCr and Ti6Al4V alloys. The hypothesis was that cyclical exposure of the unused powder to elevated temperatures in the printing chamber during printing might cause microstructural and chemical changes in the powder that could influence the properties of the printed structures. The second aim was to evaluate the ability of individual investigation methods to detect differences in the structure and the properties of samples made using additive technologies.

## Materials and methods

The materials used in this investigation were virgin CoCr powder (Starbond CoS Powder 30) and Ti6Al4V, Grade 23 (Starbond Ti5 Powder 45), both provided by S&S Scheftner Dental Alloys GmbH, Germany. The chemical composition of each of the dental alloy powders, as declared by the producer, is given in Table 1.

**Table 1:** Chemical composition of the as-received dental CoCr and Ti6Al4V powders; all results in weight%.

CoCr	Co	Cr	W	Mo	Si	C, Fe, Mn, N
	59	27	9.5	3.5	1	< 1
Ti6Al4V	Ti	Al	V	N, C, H, Fe, O		
	89	6	4	< 1		

Solid objects made from CoCr and Ti6Al4V were fabricated by an LMP-200 3D laser printer under a protective atmosphere of 99.95% N_2_ and 99.996% Ar, respectively. New powder (referred to in the manuscript as virgin powder) and powders that had been used several times (referred to in the manuscript as reused) were investigated. The laser parameters used for each of the alloys are presented in Table 2.

**Table 2:** Laser parameters for the CoCr and Ti6Al4V 3D printed alloys, using both virgin and reused powders.

	Power [W]	Laser speed [mm/s]	Hatch distance [µm]	Layer thickness [µm]	Energy density [J/mm^3^]
CoCr	70	520	25	25	215.4
Ti6Al4V	75	520	25	25	230.8

In each cycle recycling the CoCr powder consisted of sieving it through a 50 μm sieve, whereas the Ti6Al4V powder was sieved through a 63 μm sieve in order to eliminate larger particles caused by laser spattering. After sieving, the powder was left to dry until a constant mass was achieved: in the case of the CoCr powder, the powder was dried in the laboratory atmosphere at 23 ± 3 °C, with a RH between 40 and 60%; the Ti6Al4V powder, however, which has a higher affinity for moisture binding, was dried using filters containing Al_2_O_3_ grains.

### Characterisation of the powder and as-built objects

PSD characterization using laser diffraction (SYNC Microtrac MRB) was performed on virgin and (3 cycles) recycled powder of both types of alloys. Secondary-electron imaging (SEI) and electron-backscatter diffraction (EBSD) were performed using a Zeiss CrossBeam 550 field-emission scanning electron microscope (FE-SEM) with an EDAX Hikari Super EBSD camera, using APEX software, and OIM 8.6 software for EBSD postprocessing. A 15 kV accelerating voltage and 2.0–5.0 nA probe current were used for the SE images and energy dispersive X-ray analyses (EDX), while EBSD analyses were performed on 70°-tilted samples using a 10 nA probe current and a step size of 0.5 mm. The crystallographic grain orientations are presented on inverse pole figure (IPF) maps, where various colors distinguish the orientation of a given sample direction in a crystal frame.

Patterns of the present phases were determined by means of XRD. XRD analysis was performed by PANalytical Empyrean and the data analyzed by HighScore Plus database software. XRD spectra were taken between 2θ angles 5.2° and 100° for powder specimens, and between 4° and 100° for the SLM-built specimens. For powder the step size was 0.01313°, and for the SLM specimens it was 0.0065°. The time per step was 119.85 ms for powder, and 61.2 ms for the SLM specimens.

The oxygen, nitrogen and hydrogen concentrations of structures built from the virgin and reused Ti6Al4V powder were determined according to the standard ASTM E 1409, and the hydrogen concentration by an inductar^®^ ONH Cube (Elementar) analyzer according to the standard ASTM E 1447.

### Microstructural, hardness and porosity measurements

The porosity of the as-built structures was investigated classically by 2-dimensional examination of the metallographically-prepared cross sections and 3-dimensional examination by microtomographic scans (XRadia MicroXCT-400, Avizo Inspect software). The porosity of the metallographically-prepared surface was determined from micrographs taken at × 12.5 magnification including the whole specimen cross-section. The microstructure was investigated metallographically using a Carl Zeiss Axio Imager Z2 on polished (for determining porosity) and etched (revealing the microstructure) samples. Ti6Al4V was etched using Kroll etchant, while CoCr alloy was etched electrolytically in 100 ml H_2_O + 4 mL HCl at 5 V for 6 s. Tomography scans were conducted at a source voltage of 150 kV, with 1600 images taken over a 360° rotation. A resolution was obtained between 2 μm (CoCr virgin specimens) and 12 μm (all other specimens), dependent on the specimen size, meaning that pores larger than 5 or 20 μm were correctly detected.

In order to study the mechanical properties, Vickers hardness tests HV 0.3 (applied force 2.94 N) and HV 10 (applied force 98.0 N) were performed on a Frank–Finotest hardness tester, according to the standard EN ISO 6507-1, in the center of a metallographically-polished transverse surface with respect to the building direction of the structures.

### Electrochemical investigation

Electrochemical tests were performed by a Gamry ref 600+ Potentiostat/Galvanostat in a three electrode cell filled with artificial saliva consisting of 0.6 g/L NaCl, 0.72 g/L KCl, 0.22 g/L CaCl_2_·2 H_2_O, 0.68 g/L KH_2_PO_4_, 0.856 g/L Na_2_HPO_4_·12 H_2_O, 0.060 g/L KSCN, 1.5 g/L KHCO_3_ and 0.03 g/L citric acid, to which 3.15 g NaF/L was added (corresponding to 1450 ppm F^-^ or 0,0763 M NaF, representing a similar F^-^ concentration as to that in mouthwash)^35^. An artificial saliva environment was selected because the investigation was designed to study the corrosion of dental alloys in an oral environment. SLM specimens of dimensions 8 × 8 × 8 mm^3^, manufactured from virgin and reused powder, were used as the working electrode, cast together with wire for electrical contact with the epoxy and sanded with SiC grinding paper up to a granulation of 600. The area of the specimens exposed was 0.64 cm^2^. Ag/AgCl was used as a reference electrode, while a graphite rod served as the counter electrode. Electrochemical measurements were performed in the following sequence: open circuit potential measurement for 6200 s, followed by linear polarization at a scan rate of 0.1 mV/s and electrochemical impedance spectroscopy (EIS) between 65 kHz and 1 mHz, with a perturbation signal of 20 mV and 7 points/decade. Potentiodynamic scans were executed starting at − 250 mV cathodically vs *E*_corr_, potential and increased in the anodic direction up to 1.2 V (CoCr) and 3.0 V (Ti6Al4V), performed at a scan rate of 1 mV/s. All electrochemical tests were performed at body temperature i.e. 37 °C.

## Results

The study involves dental powder characterization, utilizing EDX analysis, particle size distribution and various approaches to find differences in SLM manufactured objects using virgin and reused powder.

### EDX and PSD analysis

Results of EDX analyses performed on the virgin and reused CoCr powder are presented in Table 3, and analyses of the Ti6Al4V powder in Table 4. All spectra were obtained under the same conditions, such that properties of the virgin and reused powders could be compared. Regardless of the fact that light element analysis (oxygen and carbon) is strongly affected by sample contamination^36^, it is believed that the difference in oxygen concentrations (not absolute concentrations) reflects the oxidation condition of the samples analysed. At least three single spectrum measurements were performed on each powder, and the average values (with corresponding standard deviations) are presented in the tables below.

**Table 3:** Results of EDX analysis on virgin and reused CoCr powder (after sieving) and objects built from these powders. All results are given in weight %.

	%C	%O	%Si	%Cr	%Co	%Mo	%W
Virgin CoCr powder	4.29 ± 0.53	0.06 ± 0.05	0.94 ± 0.20	23.82 ± 0.67	56.73 ± 2.29	4.02 ± 0.98	10.13 ± 0.85
Reused CoCr powder	5.45 ± 1.23	3.53 ± 1.13	0.79 ± 0.10	23.06 ± 0.22	55.64 ± 3.00	3.47 ± 0.24	9.87 ± 0.47
CoCr object from virgin powder	–	–	–	25.02 ± 0.23	60.62 ± 0.67	3.94 ± 0.19	10.44 ± 0.68
CoCr object from reused powder	–	–	–	24.78 ± 0.23	60.3 ± 0.06	3.82 ± 0.04	11.1 ± 0.06

**Table 4:** Results of EDX analysis on virgin and Ti6Al4V reused powder (after sieving) and objects built from these powders. All results are given in weight %.

	%O	%C	%Al	%Ti	%V
Virgin Ti6Al4V	5.19 ± 0.63	2.02 ± 0.45	6.09 ± 0.31	84.79 ± 0.84	3.93 ± 0.22
Reused Ti6Al4V	5.73 ± 1.23	1.21 ± 1.10	4.65 ± 1.26	85.14 ± 3.02	3.87 ± 0.17
Object Ti6Al4V—virgin powder	–	–	5.76 ± 0.05	89.30 ± 0.40	4.92 ± 0.36
Object Ti6Al4V—reused powder	–	–	5.52 ± 0.12	90.3 ± 0.30	4.2 ± 0.33

In the case of the CoCr powder, it can be seen that the concentration of oxygen is significantly higher in the reused powder, although the standard deviation is high, and that the concentration of Mo is slightly lower in the recycled powder compared to the virgin powder. The oxygen concentration in the reused Ti6Al4V powder was slightly higher than that in the virgin powder, but the standard deviation is too high to confirm this. The Al concentration of the reused powder was also slightly lower. EDX analyses on the CoCr objects reveal differences in the concentration of Mo, which was slightly lower in the object made from reused powder, and W, which was slightly higher in the object made from reused powder. The Ti6Al4V object built from reused powder shows a slight reduction in the Al concentration, and an even higher decrease in the concentration of V”.

**Figure 1:**
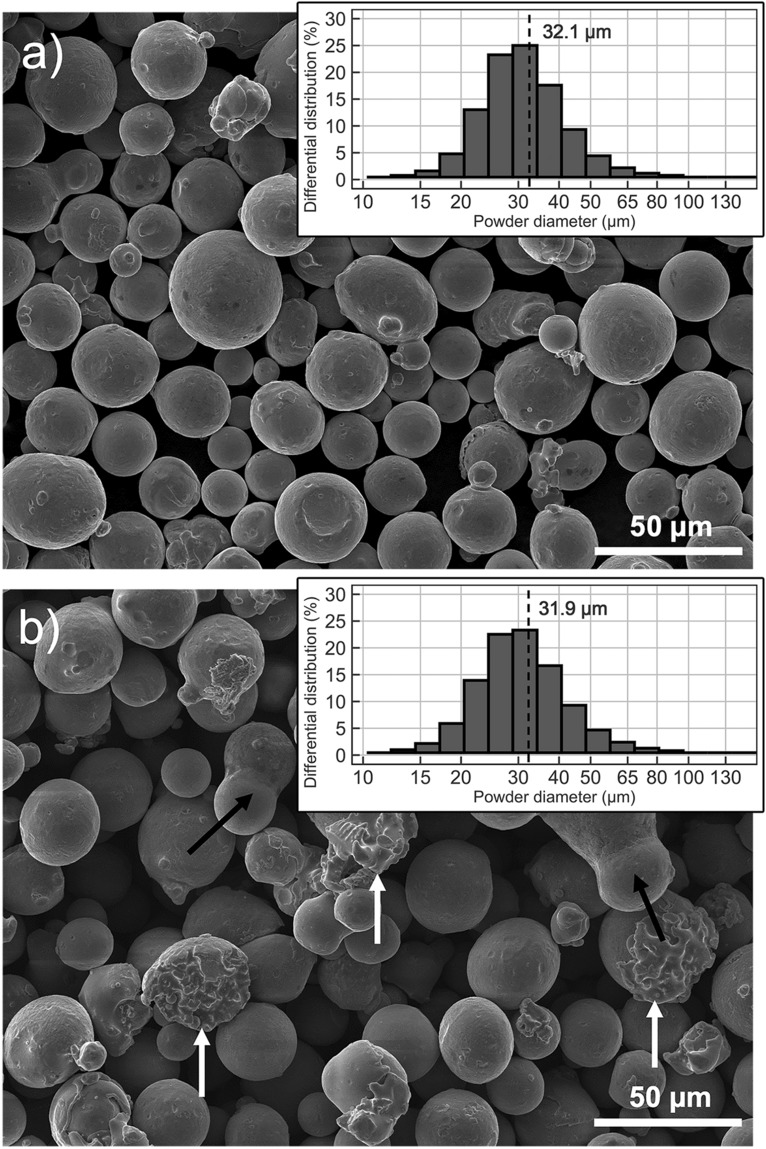
SEM images and particle size distribution of the CoCr virgin (**a**) and reused (**b**) powder.

Particle size distribution (PSD) curves for the virgin CoCr powder can be seen in the SEM photo in Fig. 1a, while PSD for the reused powder is shown in the SEM photo in Fig. 1b. The mean size of virgin and reused powder is approximately 30 µm, as declared by the producer. Recycling the powder did not influence its PSD distribution, but it did have a slight impact on the powder’s appearance: from SEM photography of the 3-times recycled powder (Fig. 1b), it can be seen that a higher number of irregularly-shaped particles (indicated by the white arrows) and larger, elongated balls (indicated by the black arrows) are present in comparison to the virgin powder (Fig. 1a).

**Figure 2:**
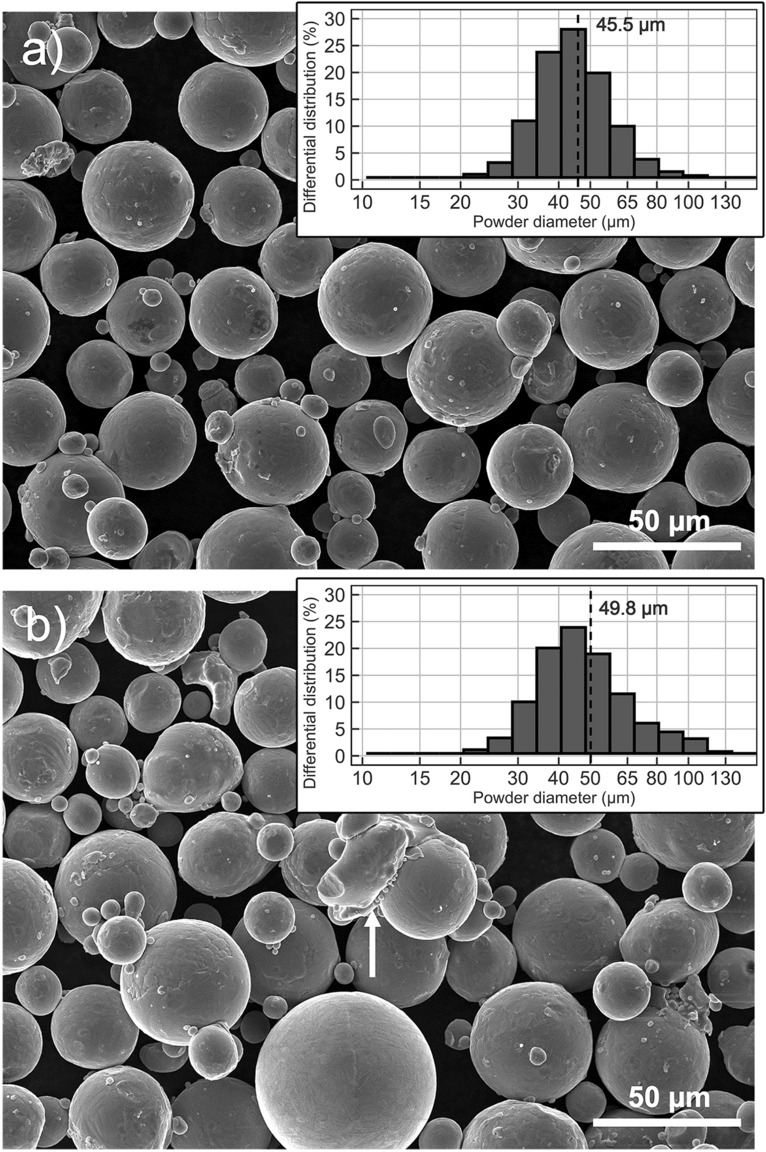
SEM images and particle size distribution of the Ti6Al4V virgin (**a**) and reused (**b**) powder.

The particle size distribution histogram for the Ti6Al4V virgin powder can be seen alongside the SEM photo in Fig. 2a, and that for the reused powder alongside the SEM photo in Fig. 2b.

According to the manufacturer, the mean size of the Ti6Al4V powder particles is the same for both, at approximately 44 µm. In the reused powder, 42% of the particles are larger than the mean, while the same proportion is only 34% in the virgin powder. A shift towards larger powder diameters can be seen in the reused Ti6Al4V powder from the curves in Fig. 2a. The SEM image of the reused powder in Fig. 2b shows the presence of irregularly-shaped particles, most probably caused by laser spattering (see the white arrow), which shifts the PSD towards higher diameters.

### XRD diffraction patterns analysis

The aim of using XRD analysis was to compare the spectra of virgin and reused powder and the SLM fabricated objects in order to find possible differences.

**Figure 3:**
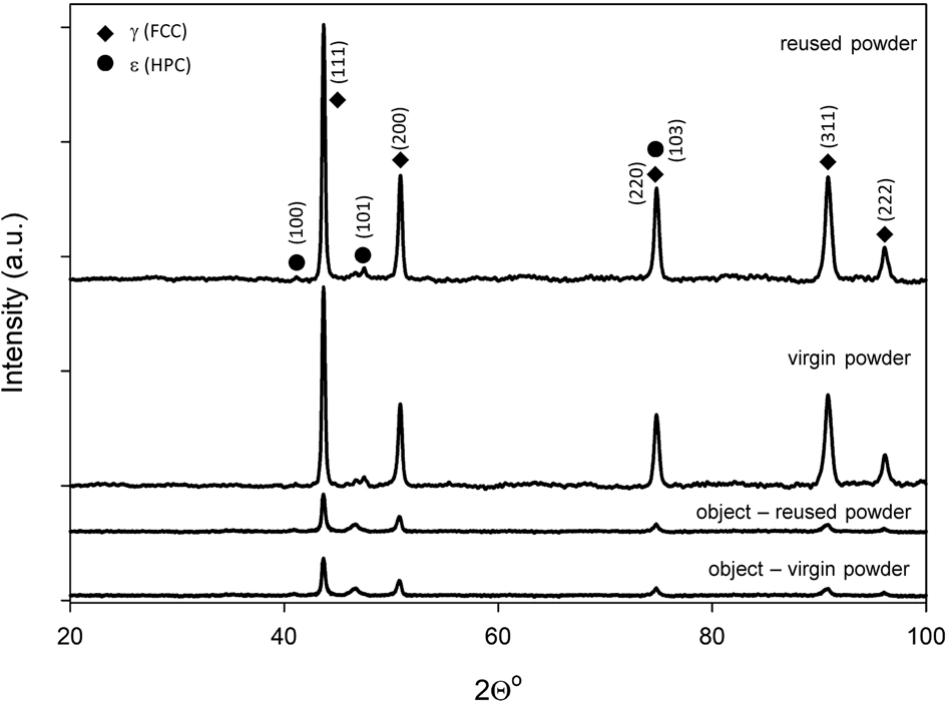
XRD spectra for the virgin and reused CoCr powders and the 3D objects printed from them.

**Figure 4:**
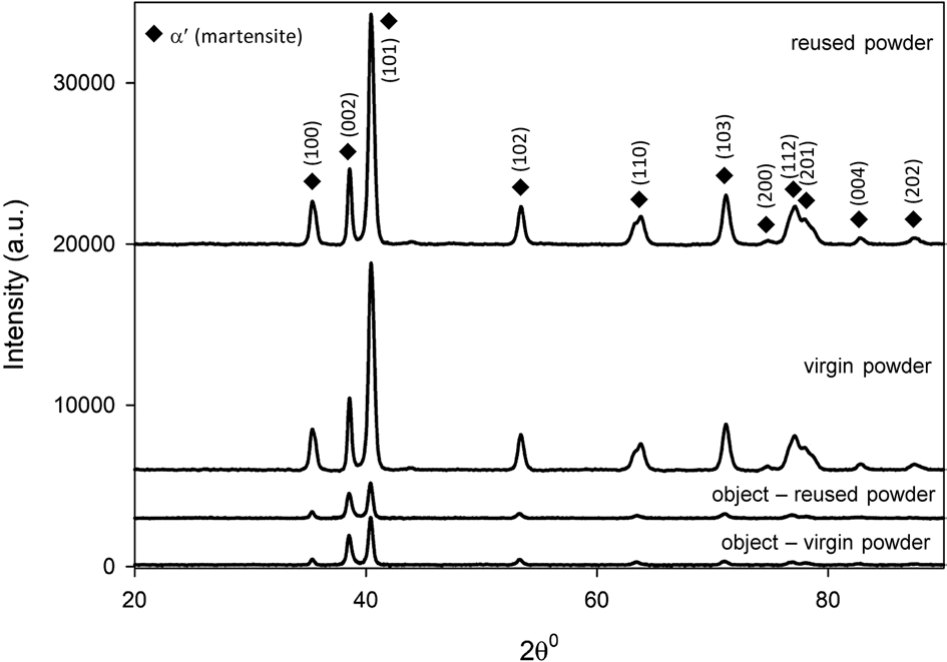
XRD diffractogram for the virgin and reused Ti6Al4V powders and the 3D objects printed from them.

XRD spectra for the CoCr alloys fabricated from virgin and reused powder, as well as for specimens of the virgin and reused powder, are presented in Fig. 3. Ti6Al4V spectra for elements fabricated from virgin and reused powder, as well as for both types of powder, are presented in Fig. 4.

The diffractogram peaks of all the CoCr powder and 3D printed objects investigated show that in both types of powder, virgin and reused, as well as the objects built from both powders, the metastable phase γ (FCC) mainly occurs. A peak of ε martensitic phase (HPC] is also observed. There is no significant difference between either the intensity or shape of all the peaks detected (Fig. 3).

The diffractogram peaks of the Ti6Al4V powder and 3D printed objects show only the presence of martensite (α′). The martensitic phase α′, which is developed due to high cooling rates for the printed object during the solidification process, cannot, however, be distinguished by XRD—they appear at the same angles as α, but only metallographically. There is a slight difference in the intensity of some peaks of the virgin and reused powder, as well as in the peaks of the objects built from both the virgin and reused powder, but the full width of half maximum dimension (FWHM) for peaks of different intensities is comparable.

With XRD investigation we have shown that no identified phase deviations have been revealed for either the virgin and reused powders, or the 3D printed objects, in both materials investigated.

### Determining the oxygen concentration of the 3D printed Ti6Al4V alloy specimens

Oxygen is an interstitial element, which, together with nitrogen and hydrogen, easily diffuses into a titanium crystal structure at elevated temperatures, where it affects the deterioration of some of its properties (for example fatigue and ductility). Such a hazard does not exist in the CoCr alloy. The aim of measuring the oxygen concentration was to check if the oxygen uptake during the SLM process differs between objects built from virgin powder and those built from reused powder. Our investigation shows that the oxygen concentration of Ti6Al4V alloy specimens 3D printed from virgin and reused powder do not significantly differ: 0.1752 ± 0.0108% O_2_ was detected in the specimens made from virgin powder, compared to a concentration of 0.1755 ± 0.0066% O_2_ in the specimens made from reused powder. Such a difference between the oxygen measured in these specimens is negligible. In addition to oxygen, the concentrations of two more interstitial elements, hydrogen and nitrogen, were also measured. Taking into account the standard deviation, their concentrations did not significantly differ (hydrogen: virgin—40.2 ± 1.7 mg/kg, reused—45.9 ± 3.3 mg/kg; nitrogen: virgin—310.5 ± 110.9 mg/kg, reused—283.4 ± 69.2 mg/kg).

### Hardness measurements

The Vickers hardness measurements, HV 0.3 and HV 10, measured on the metallographically prepared surface of the printed objects, are presented in Table 5. Micro hardness (HV 0.3–2.94 N) was selected in order to avoid the influence of porosity on measurements. On the other hand, the higher weight (HV 10–98 N) covers a larger area or volume of material in the test, so it is therefore more representative to observe the potential influence of any defects in a specimen, such as porosity and unmelted particles, on its mechanical properties.

**Table 5:** Measurements of Vickers hardness, HV 0.3 and HV 10, for CoCr and Ti6Al4V samples printed with new and recycled powder, determined on polished surfaces by optical microscope.

	HV0.3	HV10
CoCr—virgin	394 ± 25.8	373.6 ± 8.9
CoCr—reused	409 ± 26.6	381.2 ± 5.1
Ti6Al4V—virgin	389 ± 25.6	377.0 ± 2.2
Ti6Al4V—reused	383 ± 25.5	390.0 ± 6.7

In all cases the HV10 hardness measurements are smaller than HV 0.3. Microhardness (HV 0.3) is slightly higher for the CoCr alloy specimens 3D printed from reused powder and the Ti6Al4V specimens printed from virgin powder. The HV 0.3 results, however, do not show a significant difference between the 3D objects printed from virgin and reused powder in either of the alloys examined. There is a large scatter in the HV 0.3 measurements, meaning the results obtained from objects made from virgin and reused powder are in the same range. On the other hand, in both alloys the HV 10 measurements are undoubtedly higher for the objects 3D printed from reused powder compared to those made from the virgin powder, even when taking into account the standard deviation (2% for the CoCr alloy and 3.5% for theTi6Al4V alloy).

### Porosity

**Figure 5:**
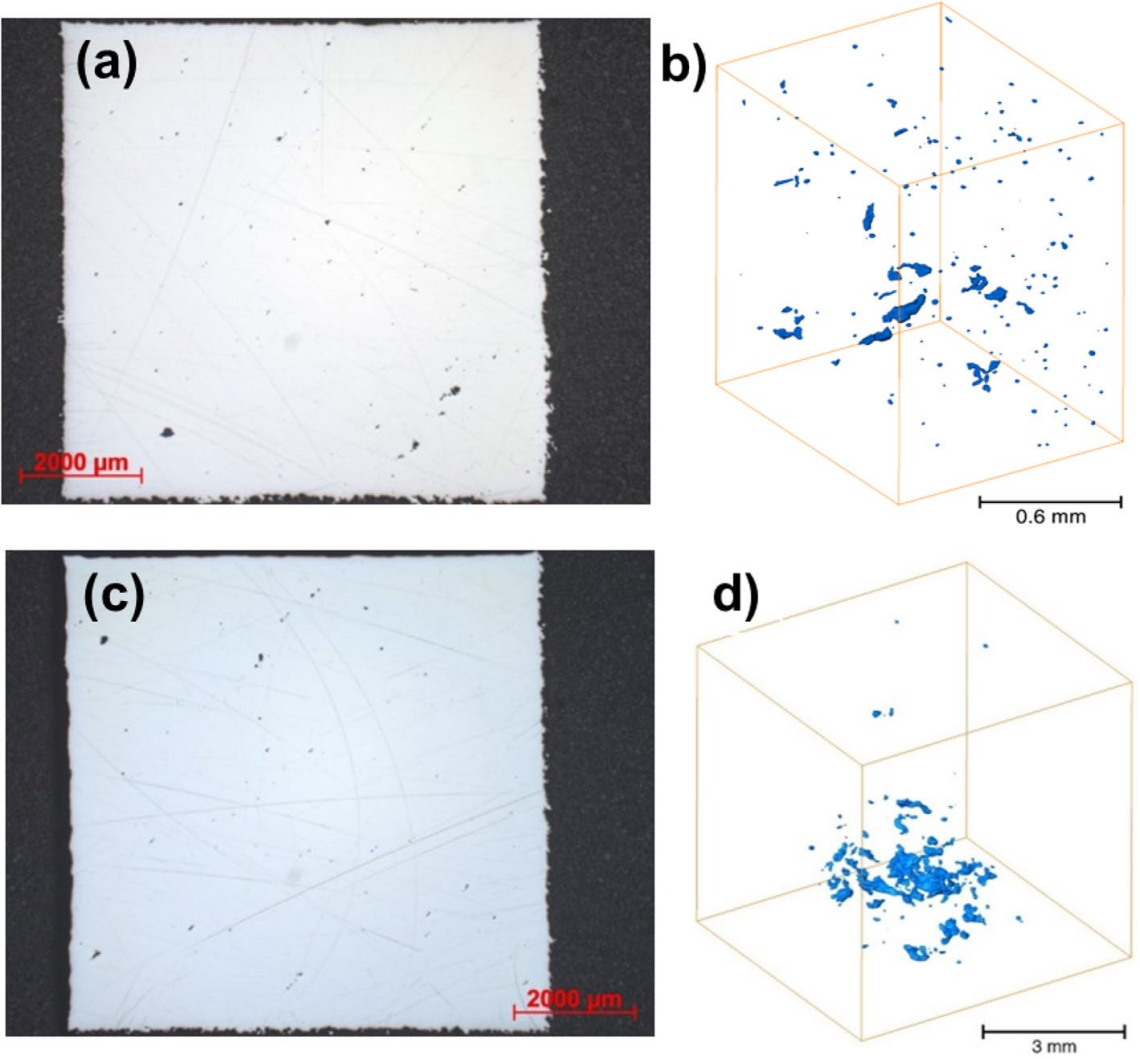
Porosity appearance of the CoCr alloy printed from virgin powder: on a 2D surface (**a**), and a cut section of the 3D microCT image (**b**), and on CoCr alloy printed from reused powder: on a 2D surface (**c**) and a 3D microCT image (**d**).

**Figure 6:**
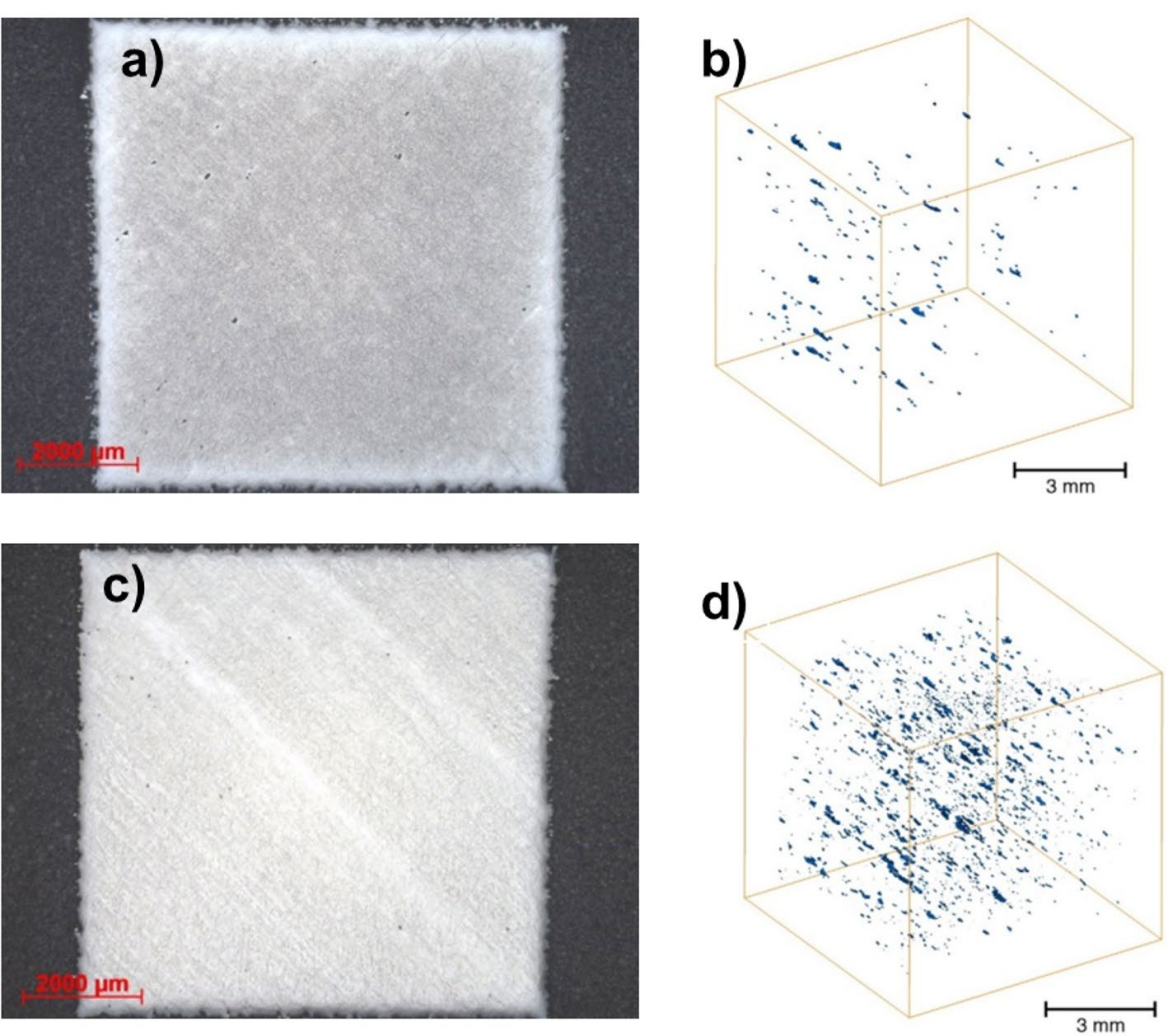
Porosity appearance of the Ti6Al4V alloy printed from virgin powder on a 2D surface (**a**) and a cut section of the 3D microCT image (**b**), and of the Ti6Al4V alloy printed from reused powder on a 2D surface (**c**) and a 3D microCT image (**d**).

The results of porosity measurements from the metallographically prepared 2D surface and by microtomography are presented in Table 6. The results were obtained from cubic specimens of roughly 8 × 8 × 8 mm^3^ in dimension, with the CoCr—virgin specimen further cut into a smaller 2 × 2 × 4 mm^3^ prism. The porosity was calculated as the volume/surface area of the detected pores divided by the total measured volume/surface area of the 3D printed cube (microCT/metallographic method, respectively). The pores were smaller and more refined in the CoCr virgin specimen, and the density and thickness of the original specimen was too large to obtain accurate porosity results. Figure 5 shows the porosity appearance of the CoCr alloy printed from virgin powder on a 2D surface (a) and a 3D microCT image (b), as well as for the same alloy printed from reused powder (2D surface—c and microCT image—d). The bottom left side of the 3D volumes presented were closest to the printing bed. The same results for the Ti6Al4V alloy are presented in Fig. 6.

**Table 6:** Porosity measurements for the CoCr and Ti6Al4V specimens printed with virgin and reused powder, determined on polished surfaces by optical microscope and microtomography.

	Porosity—cross section surface [%]	Porosity—whole specimen (microtomography) [%]
CoCr—virgin	0.13	0.05
CoCr—reused	0.21	0.19
Ti6Al4V—virgin	0.19	0.02
Ti6Al4V—reused	0.06	0.07

There is a discrepancy between the porosity results obtained from the 2D surface cross section and by 3D volume examination (microCT), as presented in Table 6. The tomography porosities were generally lower in all specimen types. In the CoCr specimens, the porosities obtained were higher in the reused powders compared to the virgin powder with both types of analysis, while for the Ti6Al4V specimens the results were inconclusive. Namely, the resolution of the 3D analysis was either 2 μm (CoCr virgin specimen) or 12 μm (other specimens) for the tomography data, while the resolution of the 2D surface porosity measurement was approximately 16 μm. Additionally, microtomographic examination made the visualization and analysis of the size and quantity of pores possible in 3D over the entire scanned volume (Figs. 5b,d, 6b,d). The volume of the pores was roughly 16 mm^3^ for the CoCr virgin specimens and 512 mm^3^ for the other specimens. 2D metallographic analysis was carried out on a randomly chosen cross-section, which was then taken as a representative sample of the entire specimen and thereby affects a greater possible inaccuracy.

**Figure 7:**
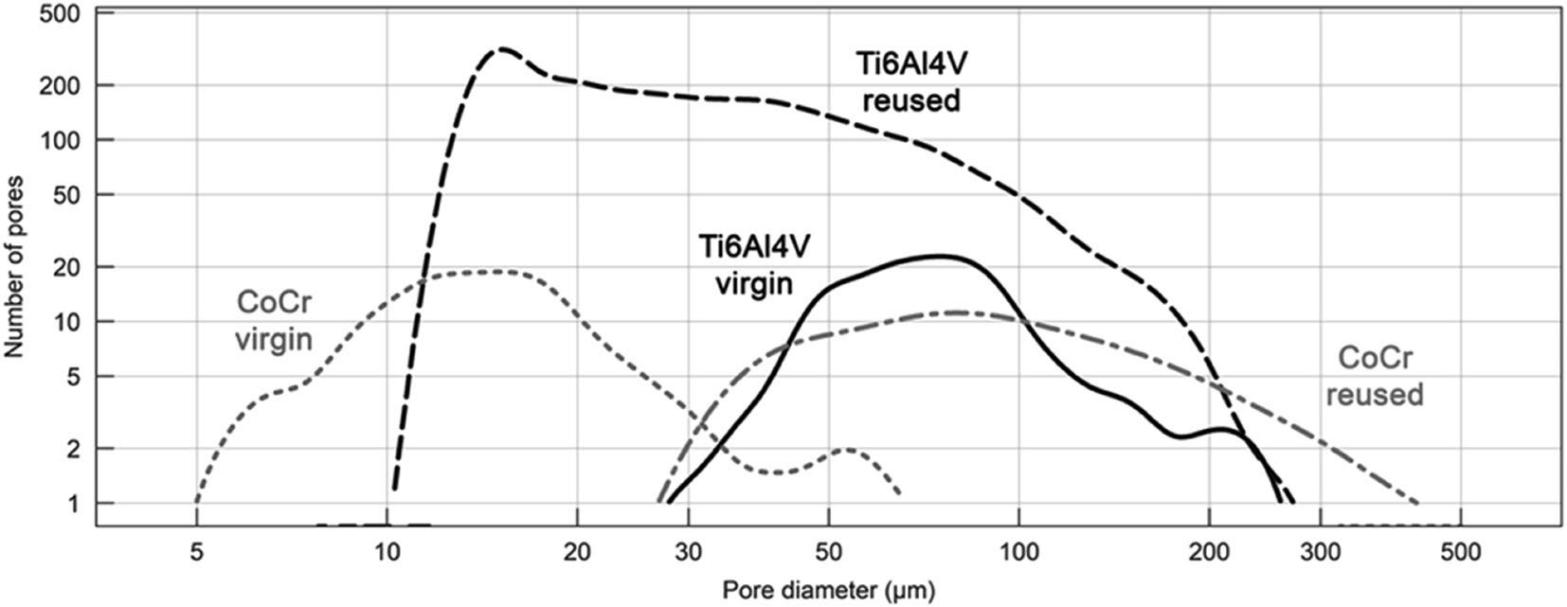
Comparison of pore diameter distribution for CoCr and Ti6Al4V alloys made from virgin and reused powder, represented as the total number of pores. Pore diameter distribution for the virgin CoCr material is not shown as no pores were detected through microtomographic scanning.

Comparing the pore diameter distribution curves shown in Fig. 7, it can be observed that the object printed from CoCr virgin powder had the smallest and most refined pores, ranging from 5 to 60 μm. The pores were equally distributed across the volume scanned (Fig. 5b). Conversely, the CoCr specimens made from reused powder had the highest number of pores above 200 μm in diameter, which were primarily located towards the bottom of the specimen where the 3D printing bed was located (Fig. 5d). This would also explain its higher total porosity compared to all the other specimens (Table 4).

Figure 6b,d show that in the Ti6Al4V material the pores were more equally distributed across the volume, with more elongated pores, in specimens from both the virgin and reused powder. Virgin specimens made of Ti6Al4V had the highest number of pores in the 40 and 150 μm range (Fig. 7), which was comparable to the specimens made from reused CoCr. Reused Ti6Al4V material had the highest number of pores up to approximately 200 μm in diameter. In the Ti6Al4V the difference between the virgin and reused powder material was especially large for pores smaller than 40 μm.

### Microstructural and EBSD examinations

**Figure 8:**
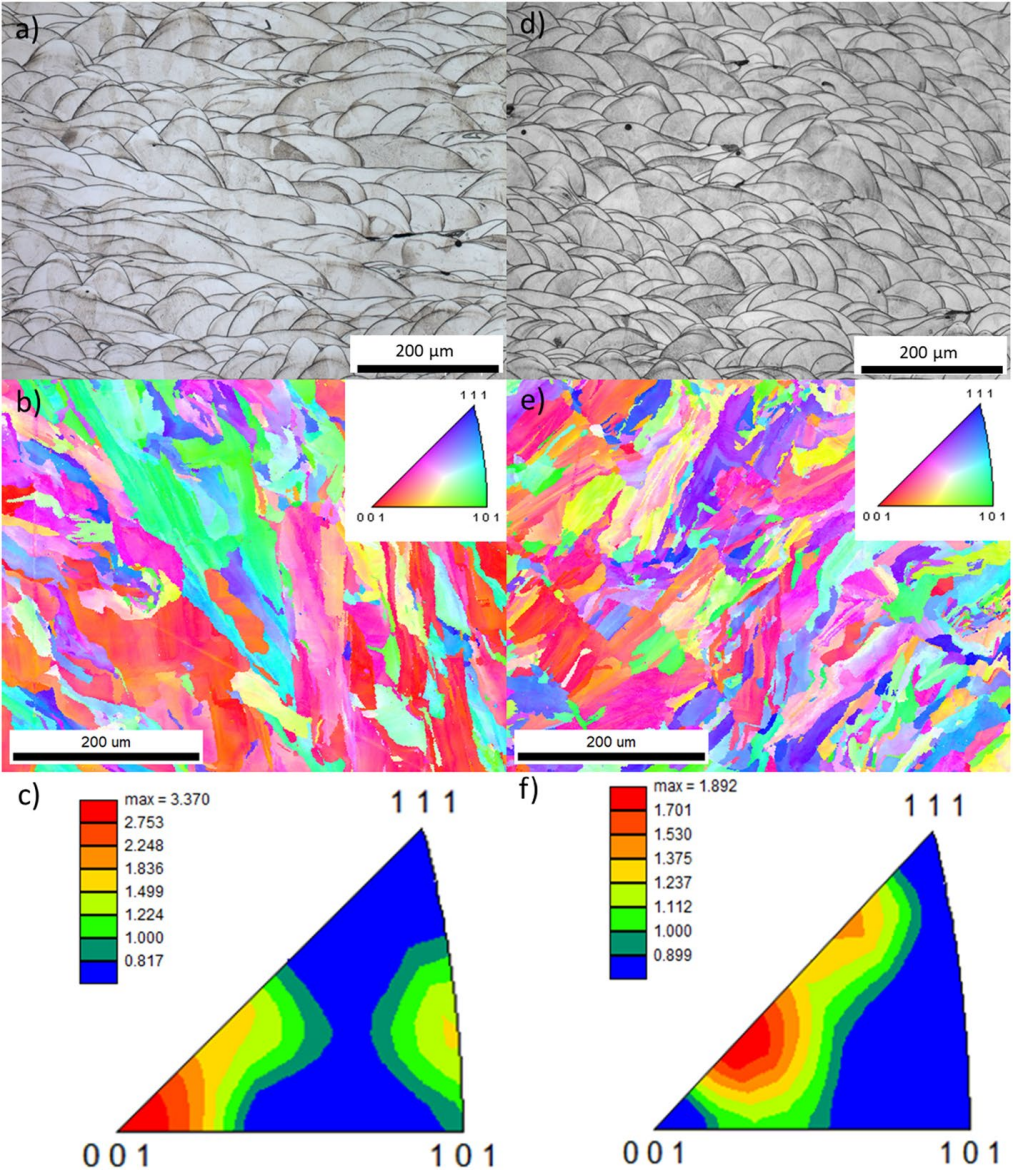
Optical micrographs of the etched microstructure, with the melting pools visible (in CoCr printed from virgin powder (**a**) and the reused powder (**c**)) EBSD results and orientation maps (in CoCr printed from virgin powder (**d**) and the reused powder (**e**)) and inverse pole figures (IFP) in [001] direction (in CoCr printed from virgin powder (**c**) and the reused powder (**f**)).

The microstructural properties of CoCr and Ti6Al4V alloys are unique for 3D printed materials. The characteristic morphology of the laser beam melting process can be observed from the metallographic images of the CoCr alloy built from both virgin (Fig. 8a) and reused powder (Fig. 8c). The (etched) microstructure consists of visibly overlapping layers and significant melting pools, which were generated by the passage of the laser beam during the selective melting and solidification of the powder. Small cellular features are present within a single melting pool, which were generated by rapid cooling during its solidification. The width of melting pools of CoCr alloy built from virgin powder is higher, and subsequently the density of melting pools of CoCr alloy built from reused powder is also higher. There are no visible differences in the cellular microstructure of the CoCr alloys compared^5,37^. Figure 8b,e show orientation maps of samples manufactured from virgin and reused powder, and Fig. 8c,f inverse pole figures (IPF) in normal direction where it can be seen that the grains are randomly oriented.

**Figure 9:**
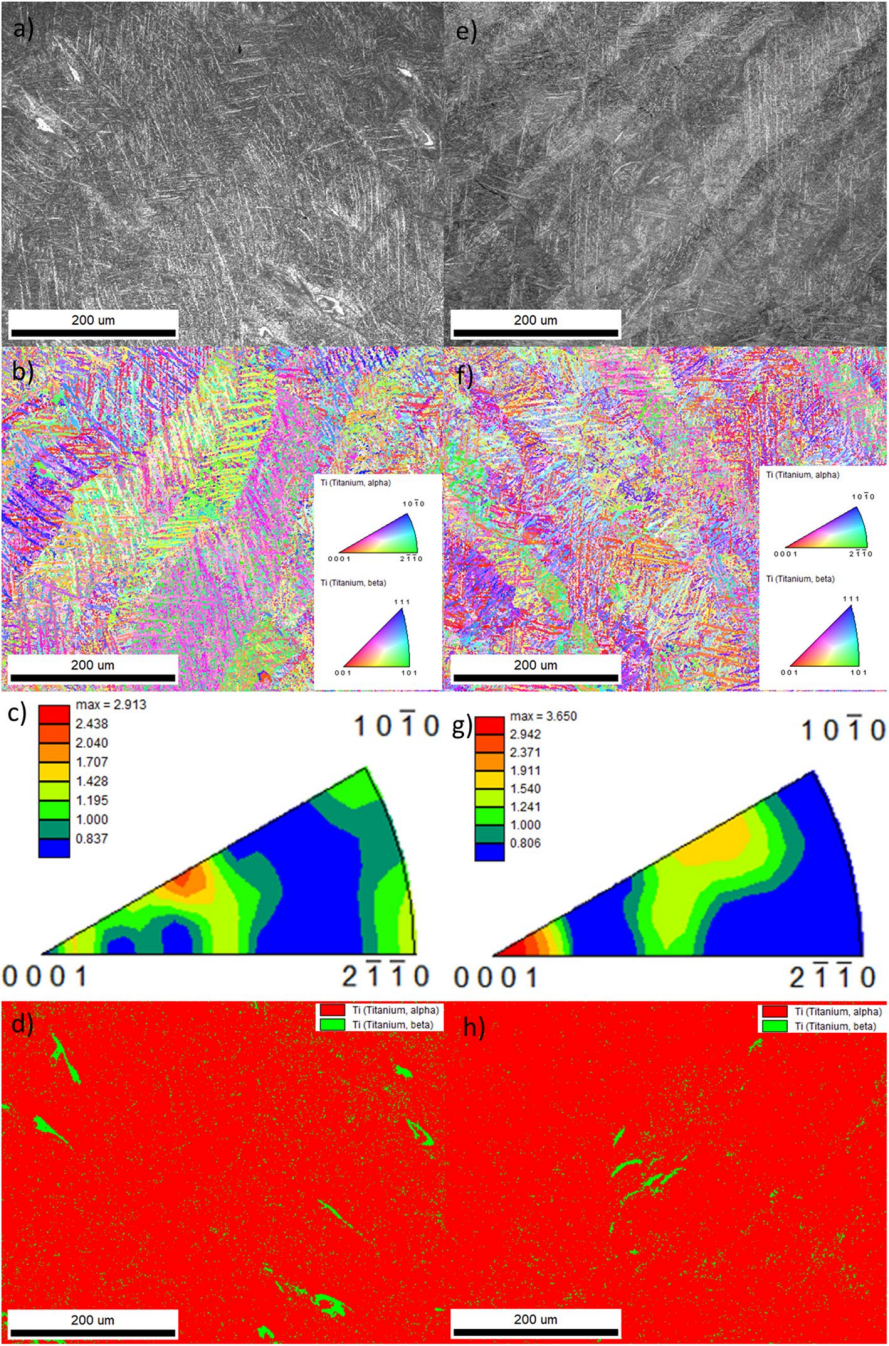
SEM micrographs of Ti6Al4V printed from virgin (**a**) and reused powder (**e**); EBSD orientation maps for the virgin (**b**) and reused (f) powder, IPF in [001] direction for the virgin (**c**) and reused (**g**) powders, and phase distributions for the virgin (**d**) and reused (**h**) powders.

The microstructures of the Ti6Al4V alloy printed from virgin (Fig. 9a) and reused (Fig. 9e) powders are similar. Columnar grain growth is observed in the SLM fabricated microstructure of the Ti6Al4V alloys made from both virgin and reused powder. The microstructure within elongated (epitaxial) prior β-grains contain fine acicular α′ needle-like martensite laths under different inclinations, due to the high cooling rate during solidification from the laser melt pool temperature^38^, but EBSD also detected a small amount of β-phase, which was slightly higher in the object printed from virgin powder (Fig. 9d—green (β) phase—4.6%) compared to the one from reused powder (Fig. 9h—green (β) phase—2.4%). Orientation maps of samples manufactured from virgin and reused powder can be seen in Fig. 9b,f, respectively, and IPF for both powders in Fig. 9c,g. No significant preferential orientation of the grains was found.

### Electrochemical properties of the CoCr and Ti6Al4V alloys

**Figure 10:**
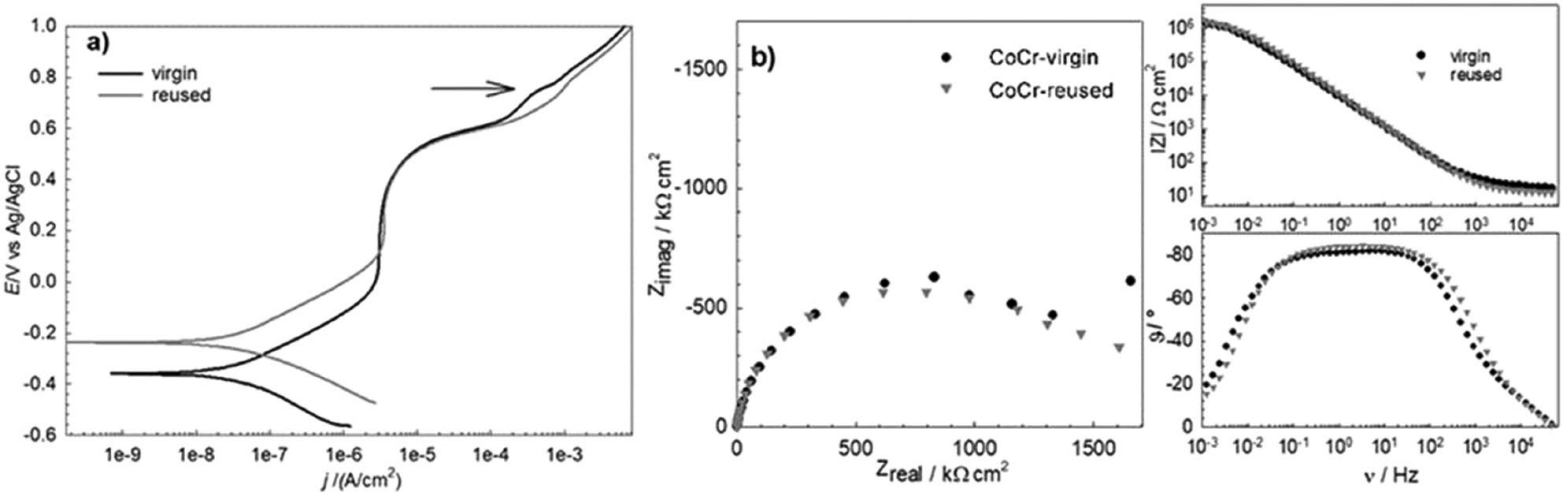
Potentiodynamic polarization curves (**a**) and Nyquist and Bode plots (**b**) for the CoCr alloys 3D printed from virgin and reused powder in artificial saliva with NaF.

**Figure 11:**
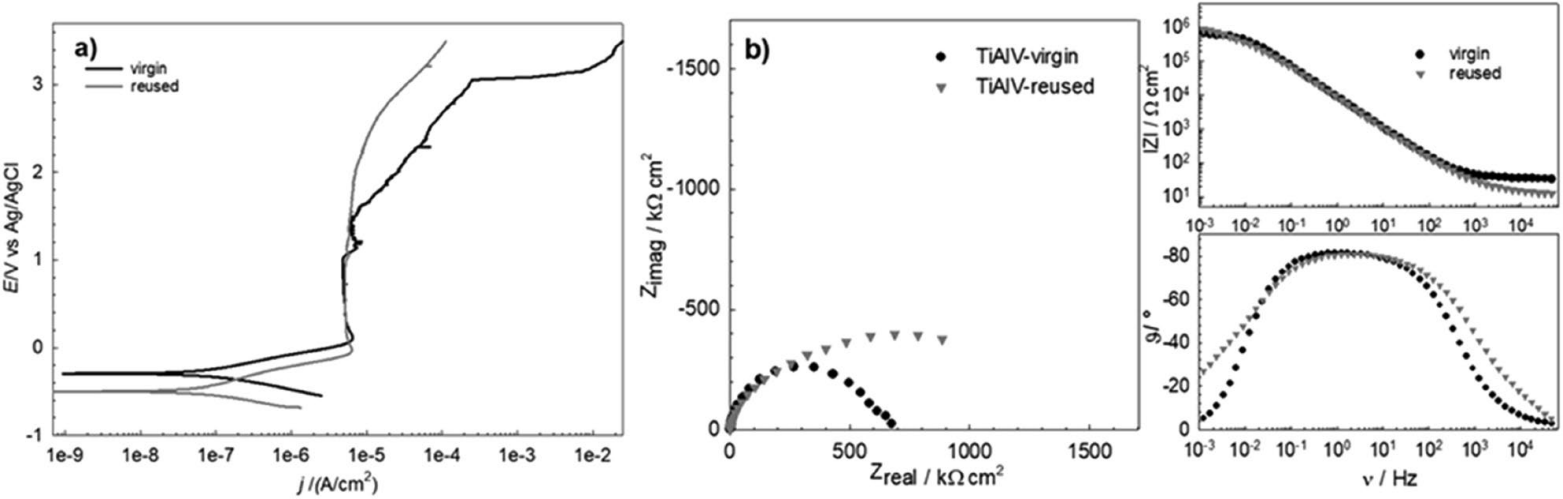
Potentiodynamic polarization curves (**a**) and Nyquist and Bode plots (**b**) for the Ti6Al4V alloys 3D printed from virgin and reused powder in artificial saliva with NaF.

Corrosion susceptibility of SLM-fabricated samples from virgin and reused CoCr and Ti6Al4V powder was electrochemically investigated in artificial saliva containing fluoride ions at body temperature. Potetiodynamic scans and electrochemical impedance spectroscopy were used. Potentiodynamic curves for the CoCr and Ti6Al4V alloys are respectively plotted in Figs. 10a and 11a, and Nyquist and Bode plots are presented in Figs. 10b and 11b. Corrosion parameters derived from the electrochemical measurements are collected for all the printed materials investigated in Table 7.

**Table 7:** Electrochemical parameters obtained from the different types of electrochemical measurement (EK—corrosion potential, LPR—linear polarization resistance, PD—potentiodynamic polarization and EIS—electrochemical impedance spectroscopy).

	EK	LPR			PD			EIS
	*E*_corr_ [mV]	*E*_corr_ [mV]	*j*_corr_ [nA/cm^2^]	*R*_p_ [MΩ m^2^]	*E*_corr_ [mV]	*j*_corr_ [nA/cm^2^]	*E*_b_ [V]	|Z| MΩ cm^2^
CoCr—virgin	− 341	− 341	34.3	0.759	− 358	39.4	541	1.13
CoCr—reused	− 241	− 239	37.5	0.695	− 237	32.5	552	1.05
Ti6Al4V—reused	− 455	− 457	51.8	0.503	− 498	65.8	–	0.577
Ti6Al4V—virgin	− 329	− 303	74.6	0.35	− 295	76.4	3000	0.436

The potentiodynamic curves for the virgin and reused CoCr powders showed similar behavior for both samples. Namely, in the anodic part of the PD curve, the corrosion current first increased with potential, then a stable and constant corrosion current was measured in the passive region (Fig. 10a). In the transpassive region (denoted by an arrow for the virgin powder), a lower current is observed for virgin powder. The corrosion potential, *E*_corr_, for the virgin powder is lower, thus the passive region is wider. It can be seen from the Nyquist and Bode plots in Fig. 10b that the impedance responses are similar for both specimens. Results of the absolute impedance, |Z|, and polarization resistance, *R*_p_, however, are slightly higher for the specimens made from virgin powder, meaning that the corrosion resistance of the specimen from virgin powder is slightly better in comparison to the sample fabricated from reused powder (Table 7).

It can be seen from the potentiodynamic polarization curves for Ti6Al4V (Fig. 11a) that the corrosion potential for the specimen built from virgin powder shifted to more positive values (− 0.295 V) than the *E*_corr_ of the specimen built from reused powder (− 0.498 V). In the passive region of the virgin powder specimen, the corrosion current density slightly increased with potential above 1.2 V. The breakdown potential, *E*_b_, for the virgin powder specimen occurred at a very positive potential, above 3.0 V. At this potential the specimen from reused powder did not reach breakdown. It can be seen from the impedance measurement (Fig. 11b) that the overall impedance for reused powder specimens is larger than that for the virgin powder specimens, which is an indication of better passivity. The polarization resistance obtained from linear polarization and the lower corrosion current density value confirm that the specimen from reused Ti6Al4V powder had better corrosion resistance overall.

## Discussion

In this research study, 3D printed objects for dental applications, made from virgin and reused CoCr and Ti6Al4V powder, were analysed and compared. The influence on the mechanical properties, microstructure and electrochemical behavior of the 3D printed specimens was studied. The identification of differences was sought through the use of different approaches and methods.

The most obvious differences observed relate to the PSDs of the Ti6Al4V alloy, as described in chapter 3.1., the porosity, as described in chapter 3.5., the microstructure, as described in chapter 3.6, and differences in the electrochemical measurements, as described in chapter 3.7.

The research study confirmed that the CoCr specimen built from the reused powder exhibited a higher porosity. Furthermore, microCT analysis revealed a higher porosity (%). It can be seen from this type of analysis (Fig. 7) that the size of the pores also differs i.e. it strongly shifted towards larger pore diameters. If only considering the PSD analysis of the powder, this is a surprise. If we look at the actual condition of the powder (SEM observation), however, we can see that larger particles have formed in previous printing cycles as a result of laser spattering and balling. The resulting particles have a more elongated shape, meaning they very probably slipped through the holes in the sieve, which were 50 µm in diameter. Additionally, EDX analysis showed a higher presence of oxygen in the reused CoCr powder, which may have led to oxidation porosity^9^. In the case of the Ti6Al4V powder, the PSD curve shifts towards larger diameters in the reused powder, which, to a certain extent, can be explained by the higher proportion of larger particles in the reused powder compared to the virgin powder. This is in agreement with the study by Ardila et al.^13^, who reported that this shift in the PSD curve was due to the aggregation of undesired particles. Other authors^16,17^, on the other hand, have observed a narrowing of the PSD in reused powder compared to that of virgin powder, which has been attributed to the detachment of satellite particles during printing. A narrowing of the PSD could, however, also reduce the density of the printed object, since the powder is spread across the print platform and the diameters of powder particles present are not sufficiently varied to fill all the voids^16,17,21^. The most likely mechanism of Ti6Al4V forming larger particles was the adherence of unmelted powder (particles with a smaller diameter) to splashing particles formed during laser spattering^9^. These larger powder particles cannot be completely melted with by the laser process and subsequently, as a direct result of the incomplete melting, the porosity in the specimens built from reused powder was higher. A comparison of EDX analyses of the virgin and reused Ti6Al4V powder did not reveal a major difference in oxygen concentration. It is believed that the oxidation of laser spatters formed during Ti6Al4V printing is negligible^10^.

It was seen through 2D surface measurements that in the case of the Ti6Al4V alloy the porosity was higher in the specimens built from virgin powder compared to those made from the reused powder, while the opposite result was obtained by microtomography. Microtomography, however, is a far more accurate type of analysis as it captures a much larger volume of the sample in the analysis compared to the porosity measurement of a 2D surface. It is obvious from the porosity measurements determined by the two different methods (i.e. 2D surface micrographs and 3D computed microtomography), that the results of each technique could lead to different conclusions.

It was expected that the hardness measurements would reflect differences in the porosity and microstructure of the built objects being compared^39,40^. There was no significant difference between the hardness measurements of specimens built from virgin and reused powders. Rather than high standard deviations, as seen with the micro hardness measurements (HV 0.3), however, minor differences were observed, as explained below. For the Ti6Al4V specimens this result is in line with the oxygen measurements, which are almost the same for objects built from both types of powder. If the atmosphere in the chamber is not sufficiently protective, the increased oxygen (or other interstitial elements such as nitrogen and hydrogen) that diffuses into the titanium lattice at elevated temperatures during the SLM process causes an increase in hardness^30^. A slight decrease was seen in the Al and V content of the Ti6Al4V powder, both in the reused powder and in the as-build object, with the decrease in the V concentration being higher. Here, V is a β-phase stabilizer that contributes to a higher hardness. EBSD analysis of the Ti6Al4V object built from reused powder shows that the amount of β-phase is lower, which could be the reason for the slightly lower microhardness of this material (HV0.3). Conversely, some differences in the HV 10 hardness were observed, being higher in the Ti6Al4V alloy than the CoCr alloy. The higher hardness measured when using a reused powder is the opposite of expectations based on the porosity measured, given that a higher porosity was observed on specimens built from the reused powder, and also based on results from the literature^41^. The slightly higher hardnesses measured on Ti6Al4V built from reused powder can, however, be explained as a result of the slightly higher concentration of oxygen and hydrogen in this material in comparison to the alloy built from virgin powder. On the other hand, slightly higher values of the CoCr specimen built from virgin powder could be a consequence of the higher number of melt pools and thus the higher density of boundaries, but also because of the significantly higher oxide measured in the reused CoCr powder. In addition to oxygen, differences were also observed in the amount of other alloying elements in the virgin and reused powder of the two different alloys. With the CoCr alloy, a slightly decreased Mo concentration was seen in the recycled powder, as well as the built object, which is probably caused by the volatility of this element during laser spattering and the printing process. In general, the increased concentration of molybdenum in the CoCr alloy increases the hardness and improves the corrosion properties^42^. On the other hand, an increase in the W concentration was seen in the object built from reused powder. Similar to molybdenum, Wolfram strengthens the solid solution and consequently increases the hardness^43^. It could be assumed that this is the cause of the higher hardnesses obtained in the CoCr objects built from reused powder. By comparing the porosity measurements on the 2D (metallographic) surface and the built specimen, it could be concluded that the porosity is not necessarily evenly distributed across the sample. This discrepancy could be attributed to the fact that hardness is measured on a few points on a 2D plane and is therefore not completely representative of the whole specimen.

It can be concluded from the XRD diffractograms of the virgin and reused powder, as well as for those of the solid objects printed from virgin and reused powder, that there were no differences in the phases detected. The only difference observed was in the peak intensities of the Ti6Al4V powder and the built objects. The difference in intensity of some of the Ti6Al4V peaks in the built objects probably originated from the high anisotropy of this alloy and the crystal orientation consequently preferred, where even small imperfections in preparation of the sample being observed (for example an inclination from the build direction of the specimen) might cause such a difference in the diffractogram^44–46^. According to our knowledge slightly lower peak intensities of the virgin powder in this alloy could only be explained by the amount of powder in the holder—with a lower amount leading to a lower intensity. This might somehow be related to the PSD distribution, but it is difficult to explain. It seems that the distribution of particles sizes over a wider range enables holder spaces to be better filled with powder, similar to as has been explained previously^47^.

Electrochemical investigation of the CoCr alloy did not reveal any significant differences between the corrosion resistance of the specimens printed by the virgin or reused powder. Electrochemical measurements were conducted in artificial saliva with added fluorides. In such an environment the CoCr alloy instantaneously forms a passive layer in the presence of oxygen. A wider “pseudo” range and passive region is observed for the virgin powder, with the corrosion potential (*E*_corr_) also occurring at lower potentials, indicating a wider passive range on the surface for the virgin powder. A transpassive peak is also observed for the virgin powder, which has previously been shown to occur in a corrosive solution consisting of phosphates due to the incorporation of Cr (VI) into a passive film^48^ at higher potentials. With respect to the corrosion behavior of the Ti6Al4V alloy, it was shown that specimens made from reused powder showed lower current densities in the anodic region compared to those made from the virgin powder. The microstructure is identical, whether examined metallographically or by XRD, with the only difference being the quantity of pores. It is known from the literature^49–52^ that heat treatment of a near α titanium alloy such as Ti6Al4V increases the corrosion resistance of both materials due to changes in the microstructure: β phase, which is partially transformed from α and α’ during exposure to elevated temperatures, is more resistant to corrosion than previous phases^53^. The presence of β phase was not, however, detected by either of the methods (metallography or XRD) in the Ti6Al4V specimens investigated in this research. One of the most probable explanations for this is the fact that a variation in porosity across the surfaces influences the corrosion behavior. A similar assumption could also be made to explain potential differences in the corrosion behavior of the CoCr alloy.

## Conclusion

The present study investigated the effect of reusing powder when fabricating dental assets from CoCr and Ti6Al4V alloys. The results showed that reusing CoCr and Ti6Al4V powder in the SLM process only has a minor influence on the microstructural, mechanical and corrosion properties and that there is no significant risk of the biocompatibility and mechanical properties deteriorating. The following conclusions can be drawn:

1. Particle size distribution measurements showed that virgin and reused CoCr powders are very similar in size. Larger powder particle sizes were measured in the Ti6Al4V reused powder, which had a higher porosity compared to the virgin Ti6Al4V powder. It was shown that porosity estimation across the volume of metallographically-prepared samples was more reliable by microCT measurement than cross-sectional measurements.
2. Hardness measurements (HV 10) showed that hardness was slightly higher in the CoCr and Ti6Al4V specimens built from reused powder, probably related to the presence of oxygen: as a result of the oxidation of the recycled powder in the case of CoCr, and in the case of Ti6Al4V, due to the increased concentration of interstitial oxygen in the printed object.
3. The reason for the higher porosity of the reused powders differed according to the alloy used. In CoCr, the higher porosity is a result of oxidation of the reused powder, which leads to oxidation porosity. In Ti6Al4V, larger powder particles in the reused powder cannot be sufficiently melted by the selected laser parameters, which results in the development of pores.
4. It was not possible from basic examination of the XRD patterns to identify any phase differences between the virgin and reused powder or the objects built from each of them, whereas EBSD analysis was far more accurate and even showed a small proportion of the β-phase in the Ti6Al4V alloy.
5. The corrosion performance of SLM-fabricated objects from virgin and reused CoCr and Ti6Al4V powders showed excellent passivation in a simulated oral environment. The origin of the powder does not have a detrimental effect on the corrosion properties, which is especially important for dental applications.

## Data Availability

The datasets generated during and/or analysed during the current study are available from the corresponding author on reasonable request.
